# Hypercarotenemia in Anorexia Nervosa Patients May Influence Weight Balance: Results of a Clinical Cross-Sectional Cohort Study

**DOI:** 10.3389/fpsyt.2021.758300

**Published:** 2021-12-20

**Authors:** Sonja Lackner, Nathalie Meier-Allard, Sabrina Mörkl, Wolfram Müller, Alfred Fürhapter-Rieger, Harald Mangge, Sieglinde Zelzer, Sandra Holasek

**Affiliations:** ^1^Division of Immunology and Pathophysiology, Otto Loewi Research Center, Medical University of Graz, Graz, Austria; ^2^Department of Psychiatry and Psychotherapeutic Medicine, Medical University of Graz, Graz, Austria; ^3^Division of Biophysics, Gottfried Schatz Research Center, Medical University of Graz, Graz, Austria; ^4^Clinical Institute for Medical and Chemical Laboratory Diagnosis, Medical University of Graz, Graz, Austria

**Keywords:** anorexia nervosa, carotenoids, weight gain, body fat, adipocyte biology, treatment success, hypercarotenaemia, beta-carotene

## Abstract

**Introduction:** Anorexia nervosa (AN) can co-occur with hypercarotenemia, a clinical condition characterized by elevated β-carotene in plasma and skin tissue. Carotenoids have known anti-obesogenic effects in adipocyte biology. Thus, carotenoids may potentially play a retarding role in weight gain during the recovery of AN patients. This study evaluated the plasma carotenoid profile and subcutaneous adipose tissue (SAT) in a cohort of AN patients and normal weight (NW) controls.

**Methods:** Plasma concentrations of α-carotene, β-carotene, β-cryptoxanthin, and lycopene were determined by HPLC analysis. SAT thicknesses were measured by a highly accurate and reliable ultrasound technique. Information on dietary intakes were collected by repeated 24-h recalls.

**Results:** Sixty-two females (AN: *n* = 18, NW: *n* = 44) were included. The concentrations of β-cryptoxanthin (*p* = 0.045) and lycopene (*p* = 0.004) were significantly lower in AN patients. β-carotene levels were higher in AN patients (n.s.) and α-carotene did not differ significantly. SAT thickness was significantly lower in AN patients compared to controls (*p* < 0.001). β-carotene was significantly negative (*r*_s_ = −0.471) and lycopene significantly positive (*r*_s_ = 0.366) correlated with SAT. The correlation of β-carotene and SAT was even higher in the AN group alone (*r*_s_ = −0.742). Also, β- cryptoxanthin and the sum of provitamin A carotenoids were correlated to SAT (*r*_s_ = −0.647 and *r*_s_ = −0.746, respectively) in AN patients. Fruits and vegetable intake did not differ significantly between AN and NW but adjusted for SAT, AN patients consumed relatively higher amounts (*p* = 0.006).

**Conclusion:** Higher plasma β-carotene concentrations were associated with reduced SAT levels, most probably due to a reduced ability of the remaining adipose tissue to store carotenoids. Thus, the antiobesity effects of carotenoids might impact the treatment success of undernutrition and AN. A systemic carotenoid overload may contribute to changes in adipogenesis and metabolic capacities for energy storage. Therefore, high plasma β-carotene may be a marker of delay in weight recovery in AN patients. Interventional studies should consider including carotenoid-status in AN treatment.

## Introduction

Anorexia nervosa (AN) is one of the most lethal psychiatric diseases. It is characterized by extreme weight loss due to restrictive eating and/or purging behavior, fear of weight gain, body image disturbance and maintenance of abnormally low body weight. Although therapeutic approaches attempt to reduce the disease burden, long-term success is diminished by high relapse rates (1).

Dependent on the length of follow-up in studies and the definition, relapse rates range from 9 to 65%, with the highest relapse probability in the first 4–12 months after treatment (2). Thus, effective therapy options are urgently warranted.

Besides multiple physiological disturbances in AN patients due to reduced energy availability and malnutrition, hypercarotenemia has been reported in some AN patients. In hypercarotenemia, plasma carotenoid levels are elevated and carotenoids accumulate in the stratum corneum of the epidermis, leading to a yellowish skin appearance (3).

Carotenoids are fat-soluble secondary plant nutrients responsible for the red, orange and yellow colors of fruits and vegetables. The most prominent carotenoid is β-carotene, the most important precursor of retinoic acid. However, in human nutrition, six carotenoids (namely α-carotene, β-carotene, β-cryptoxanthin, lutein, lycopene, and zeaxanthin) have well-known antioxidant, anti-inflammatory and immunomodulatory functions in the human organism (4). The primary nutritional sources of carotenoids are fruits and vegetables (4, 5). However, due to the fat-soluble structure, they can only be utilized appropriately through lipid metabolism (5). Additionally, host-related factors such as nutritional status and body composition substantially influence the bioavailability of carotenoids (6). Vice versa, carotenoids modify immune and adipocyte metabolism. Especially for AN patients, the involvement of carotenoids in adipocyte biology (7) may be of particular interest, as carotenoids inhibit adipogenesis, reduce fat storage capacity and increase fat oxidation in adipocytes (8–10).

For hypercarotenemia in AN, different mechanisms have been proposed: First, the intake of carotenoid-containing food may be relatively high compared to energy intake and other nutrients, and the carotenoid demand may be decreased (11, 12). Secondly, disturbances in lipid metabolism such as hypercholesterinemia or acquired disturbances in carotenoid metabolism like impaired lipoprotein degradation and altered storage ability have been hypothesized (13, 14). Third, the amount of body fat may change the availability of carotenoids. As a result of the reduced amount of body fat in AN patients, the circulating carotenoid concentration may increase and carotenoids may be alternatively stored in other tissues such as the skin or the retina (15).

This study aimed to evaluate the plasma carotenoid profile and body composition in a cohort of AN patients and healthy controls, as carotenoids could potentially have a role in weight gain during AN recovery.

## Materials and Methods

### Study Population

AN patients were recruited from three psychiatric clinics in Graz, Austria. They were diagnosed by psychiatrists following the F50.0 International Classification of Diseases (ICD-10) criteria for AN (16) with a body mass index (BMI) below 17.5 kg m^−2^. All AN patients received psychiatric and nutritional treatment. For the inclusion of normal weight (NW) controls the WHO recommended BMI range of 18.5–24.99 kg m^−2^ was chosen. The control group was recruited at the university campus, word of mouth advertising, and at local sports clubs. Exclusion criteria were: acute or chronic diseases or infection, alcohol or drug abuse, major cognitive deficits, life-threatening conditions during AN, history of digestive diseases (e.g., inflammatory bowel diseases and irritable bowel syndrome), history of gastrointestinal surgery, recent treatment with antibiotics and intake of dietary supplements, pregnancy, or breastfeeding.

The study met the criteria of the Helsinki Declaration and was approved by the ethics committee of the Medical University of Graz (MUG-26-383ex13/14). All participants signed the informed consent and agreed on the anonymous use of their data.

### High-Pressure Liquid Chromatography Analysis of Plasma Carotenoids

For assessing the plasma carotenoid profile, the four carotenoids that quantitatively appear most frequently in plasma have been chosen: α-carotene, β-carotene, β- cryptoxanthin, and lycopene.

The extraction of all-trans β-carotene in EDTA plasma was done according to the manufacturer's protocol with the ClinRep® kit for β-carotene in plasma (30000, Recipe Chemicals + Instruments, GmbH, Munich, Germany). Additionally, we used external standard curves for quantification of α-carotene (50887, Sigma Aldrich, Vienna, Austria), β-cryptoxanthin (55, Carote Nature, Münsingen, Switzerland), lutein 89723 (Phytolab, Vestenbergsgreuth, Germany) and all-trans lycopene (75051, Sigma Aldrich, Vienna, Austria). The concentrations were calculated with the received standard curve with a linear fit model (y = kx + d). Either height or area was used for quantification. The method is described in more detail in Dams et al. (17).

The HPLC JASCO system (biolab, Vienna, Austria) with column oven, autosampler, and an UV detector with wavelength set at 450 nm was used. The accuracy of the measurements was proofed with the standard reference material (SRM) 968e from the National Institute of Standards and Technology (NIST Gaithersburg, USA).

### Further Laboratory Measures

Standard blood parameters for liver, kidney and thyroid function were determined by clinically established standards procedures. Blood draws were conducted in overnight fasted participants. Total cholesterol, triglycerides, and HDL-cholesterol were measured by enzymatic photometric transmission measurement (Roche Diagnostics, Mannheim, Germany) and the concentrations of LDL-cholesterol were calculated by the Friedewald's formula. The adipokines adiponectin, leptin, and leptin receptors, were determined by specific enzyme-linked immunosorbent assays (all BioVendor, Brno, Czech Republic).

### Anthropometry and Ultrasound Measurement of Subcutaneous Adipose Tissue

Body height, body weight and circumferences of waist and hip were measured in accordance with the International Society for the Advancement of Kinanthropometry (ISAK) standards (18). The BMI was calculated according to the formula BMI = body weight (kg)/body height (m)^2^ (19).

For the assessment of body fat, subcutaneous adipose tissue (SAT) thicknesses were measured by an ultrasound (US) technique (20). This method has been chosen since it was shown to accurately measure SAT in people ranging from extremely low fat layers to obese (21) whereas the application of other methods is limited in people with extreme body composition (22). SAT layers were measured at eight standardized body sites (upper abdomen, lower abdomen, erector spinae, distal triceps, brachioradialis, external oblique, front thigh, and medial calf). Ultrasound measurements were performed with a conventional US system (GE Logiq-e, General Electric) using a linear probe (L8-18i RS) operated at 8–16 MHz. The semiautomatic evaluation software (Rotosport, Stattegg, Austria) was applied for the US images evaluation. For further analysis, the value D_INCL_ was used. D_INCL_ is the calculated sum of the eight SAT thicknesses.

### Nutritive Assessment

Information on dietary intake of food groups, nutrients and energy was obtained by structured and interviewer-guided, twice repeated 24 h-recalls. The interviews were analyzed by the nutritional software nut.s^®^ (www.nutritional-software.at, Vienna, Austria) that is based on an Austrian specific food and nutrient database (23).

Dietary intake of fruits and vegetables were adjusted for energy intake and total subcutaneous adipose tissue (D_INCL_).

### Statistical Analysis

The software SPSS Statistics version 25.0 (IBM, Armonk, NY, USA) was used for statistical analysis. According to the Shapiro-Wilk test, not all of the data was normally distributed; thus, the data is presented as median and interquartile ranges (IQR) and the Mann-Whitney *U*-test was applied for group comparisons. Spearman's rank correlation coefficient r_s_ was used for the identification of correlations. For the generation of figures, the software GraphPad Prism version 9.0.0. (GraphPad Software, San Diego, CA, USA) was used.

## Results

### Study Population Characteristics, Clinical and Dietary Parameters

A total of 62 metabolically healthy females (AN *n* = 18, NW *n* = 44) aged between 18 and 35 years were enrolled in this analysis. The main study population characteristics are summarized in Table 1. While most of the routine laboratory parameters were within normal ranges in both groups, some parameters differed significantly between AN patients and NW controls: Fasting glucose was lower in AN patients [AN median 79.5 mg/dl (IQR 13), NW median 90.5 mg/dl (IQR 8)], whereas the liver parameters gamma-glutamyl-transferase GGT [AN median 19.5 U/l (IQR 13), NW median 12.0 U/l (IQR 5)] and alanine-aminotransferase ALT [AN median 23.0 U/l (IQR 16), NW median 15.0 U/l (IQR 8)] were higher.

**Table 1:** Study population characteristics.

	**Anorexia nervosa patients**	**Normal weight controls**	***p*** **-value**
***n*** **=**	**18**	**44**	
**General characteristics**			
Age (years)	22 (6)	23 (5)	0.212
BMI (kg m^−2^)	15.5 (2)	21.7 (2.3)	<0.001***
Waist circumference (cm)	60.0 (5.3)	70.6 (6.5)	<0.001***
Hip circumference (cm)	78.8 (4.8)	94.7 (9.1)	<0.001***
**Body fat measures**			
D_INCL_ (mm)	30.2 (36.1)	73.4 (41.4)	<0.001***
Leptin (ng/ml)	1.6 (2.8)	8.6 (5.3)	<0.001***
Adiponectin (μg/ml)	16.54 (8.14)	11.5 (6.11)	0.006**
**Plasma carotenoides**			
α-Carotene (μg/l)	21.2 (23.5)	26 (16.30)	0.485
β-Carotene (μg/l)	563.4 (678.6)	352.4 (341.2)	0.070
β-Cryptoxanthin (μg/l)	72.7 (90.1)	96.9 (76.6)	0.045*
Lycopene (μg/l)	18 (10.9)	27.4 (15.2)	0.004**
**Clinical chemistry**			
Fasting Glucose (mg/dl)	79.5 (13.0)	90.5 (8.0)	<0.001***
Cholesterol (mg/dl)	169.0 (57.0)	172.5 (48.0)	0.798
HDL (mg/dl)	73.0 (15.0)	80.5 (22.0)	0.214
LDL (mg/dl)	78.0 (43.0)	79.5 (23.0)	0.944
Triglycerides (mg/dl)	78.5 (54.0)	71.5 (46.0)	0.185
**Dietary intake data**			
Energy intake (kcal)	1,981.5 (1,472.0)	1,987.1 (804.0)	1.000
Fruits and Vegetables (g)	401.7 (462.4)	318.2 (356.0)	0.745
Fruits and Vegetables adjusted for D_INCL_ (g mm^−1^)	18.0 (38.1)	4.0 (5.2)	0.006**

*Data is presented as median (IQR). For group comparisons the Mann-Whiney-U-test has been applied. A p-value was considered as significant and marked with*

* *p < 0.05*,

** *p < 0.01, and*

*** *p < 0.001*.

*BMI, body mass index; D_INCL_, sum of subcutaneous adipose tissue thicknesses measured at the eight standardized body sites*.

No significant differences in total energy intake were observed and the reported total amount of fruit and vegetable intake did not differ significantly between AN patients and NW controls (Table 1). As part of nutritional treatment 10 AN patients received high-energy supplements.

Adjusted for energy intake, the amount of fruits and vegetables did not differ significantly between the groups; however, when adjusted for D_INCL_, the fruit and vegetable consumption was significantly higher in AN patients (U = 209, *p* = 0.006), meaning higher fruits and vegetable intake with regard to body fat status.

The median treatment duration of AN patients was 18 days (IQR 48.5), and the median of the reported onset of the disease duration was 1.75 years (IQR 7.5).

AN patients and NW controls reported on average no weight change within the prior 3 months [AN median: 0 kg, (IQR 10.23), NW median 0 kg (IQR 1)].

### Plasma Carotenoid—Profile

**Figure 1:**
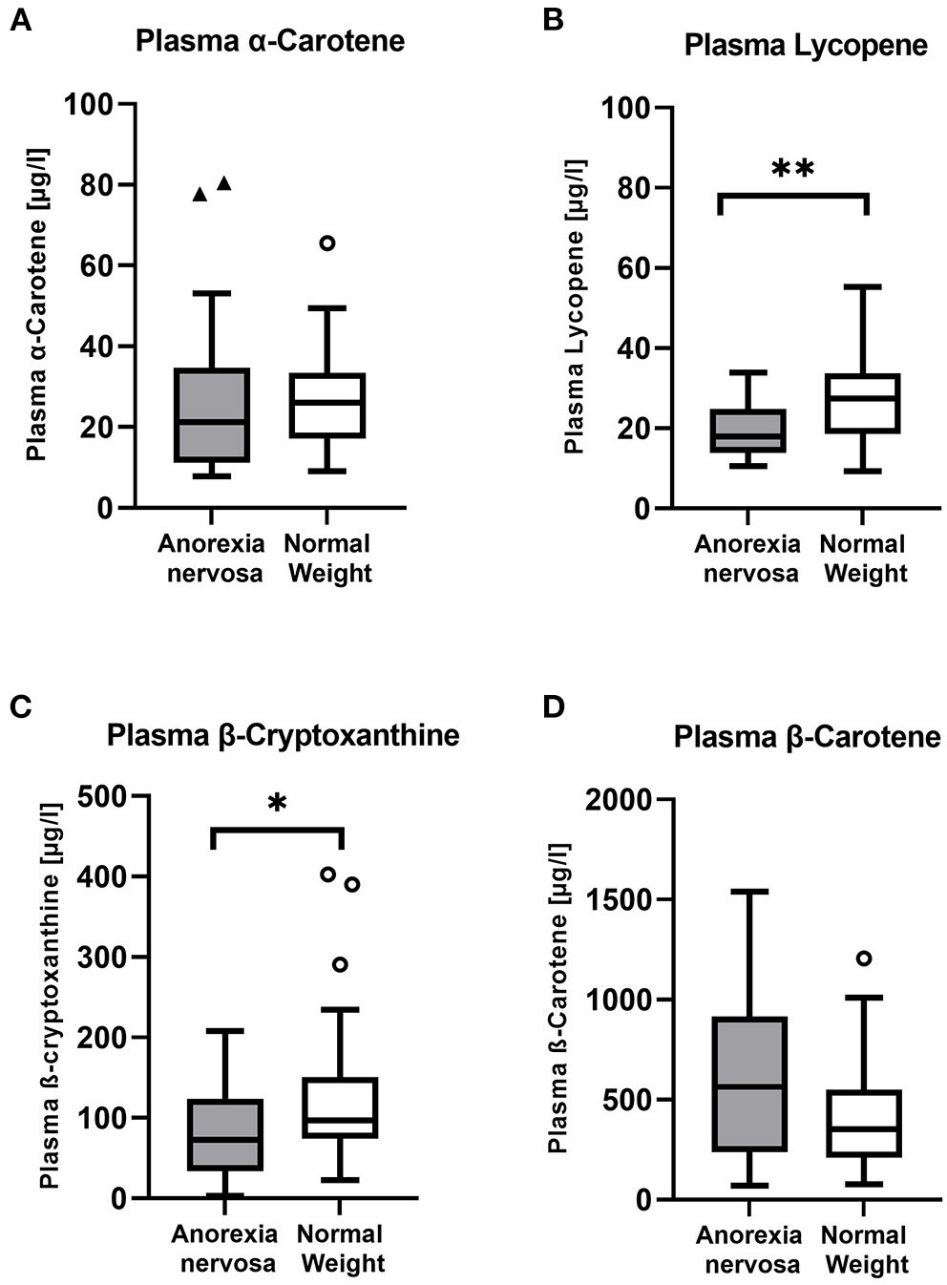
Distibution of plasma carotenoids in the study population. The plasma carotenoids lycopene **(B)** and β-cryptoxanthine **(C)** were significantly lower in the group of Anorexia nervosa patients compared to healthy normal weight controls and also the median of α-carotene **(A)** was lower in AN patients. On the contrary, β-carotene **(D)** was higher in the AN patient group. A *p*-value was considered as significant and highlighted as follows: *p < 0.05 and **p < 0.01.

The concentrations of β-cryptoxanthin and lycopene were significantly lower in AN patients compared to NW controls (U = 525, *p* = 0.045; U = 582, *p* = 0.004, respectively) (Table 1). The plasma concentration of β-carotene was substantially higher in the AN group. However, the difference was not significant. No striking differences for α-carotene could be observed. The distribution of plasma carotenoids is depicted in Figure 1.

The total amount of provitamin A carotenoids (calculated sum of α-carotene, β-carotene and β- cryptoxanthin) showed no significant difference, whereas AN had higher concentrations.

### Body Fat Measures and Subcutaneous Adipose Tissue Thicknesses

The sum of SAT thicknesses measured at the eight standardized body sites D_INCL_ was significantly lower in AN patients compared to NW controls (U = 704, *p* < 0.001) (Table 1). As previously reported, some AN patients had comparably high amounts of D_INCL_ compared with NW controls despite extremely low BMIs (24).

### Correlation of Subcutaneous Adipose Tissue Thicknesses and Carotenoids

**Figure 2:**
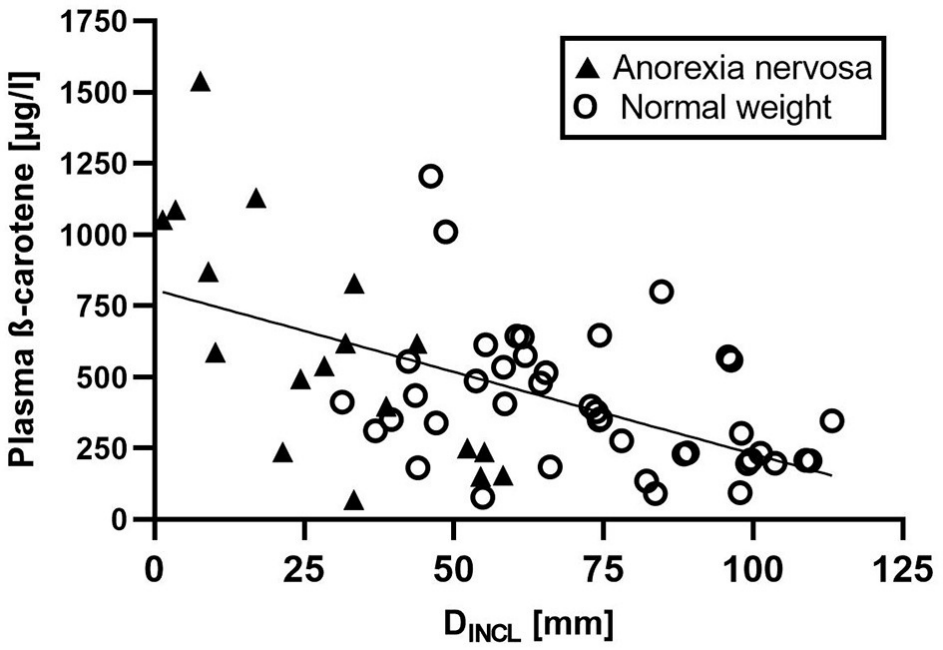
Correlation of the sum of subcutaneous adipose tissue thicknesses (D_INCL_) and plasma β-carotene. A significant negative correlation of subcutaneous adipose tissue thickness (D_INCL_) and plasma β-carotene concentration [*r*_s(60)_ = −0.471, *p* < 0.001] was obsverved in the study population. This negative correlation was even stronger in the Anorexia nervosa group alone [*r*_s(18)_ = −0.742, *p* < 0.001]. Anorexia nervosa patients are depicted as black triangels and normal weight controls are shown as white circles.

β-carotene showed a significant negative [*r*_s(60)_ = −0.471, *p* < 0.001] (Figure 2) and lycopene a significant positive [*r*_s(60)_ = 0.366, *p* = 0.004] correlation with D_INCL_ in the whole study population.

The observed correlation of β-carotene was even higher in the AN group alone [*r*_s(18)_ = −0.742, *p* < 0.001]. Interestingly, also β- cryptoxanthin [*r*_s(18)_ = −0.647, *p* = 0.004] and the sum of provitamin A carotenoids [*r*_s(18)_ = −0.746, *p* < 0.001] were significantly negatively correlated to body fat in AN patients, meaning that provitamin A carotenoids, especially β-carotene plasma levels were higher when body fat storage was reduced. Lycopene did not show a significant correlation in AN alone.

In the control group, the correlation of carotenoids and body fat remained significant for β-carotene [*r*_s(42)_ = −0.392, *p* = 0.010] and the sum of provitamin A carotenoids [*r*_s(42)_ = −0.382, *p* = 0.012], however, the correlations were much higher for AN alone.

## Discussion

Phytonutrient research proposed plausible physiological mechanisms by which dietary carotenoids impact adipocyte biology and thus influence body fat function, distribution and composition. In the case of obese individuals, reducing effects on adipose tissue and thus systemic conditions of inflammation and oxidative stress have been suggested (8, 9, 25). Since carotenoids are closely related to body fat, their investigation may reveal new insights into physiological mechanisms in AN patients during the recovery progress.

In this study, the plasma carotenoid profile of female AN patients compared to healthy NW controls was evaluated and associated with their subcutaneous adipose tissue level.

### Plasma Carotenoid Profile of AN Patients Compared to Normal Weight Females

In addition to the elevated β-carotene levels in AN patients, we found significantly decreased β-cryptoxanthin and lycopene plasma concentrations in AN patients compared to NW controls. To our knowledge, β-cryptoxanthin, α-carotene and lycopene levels have not been described previously in AN patients. The predominant dietary sources of lycopene in our study population were tomatoes and tomato products. However, the statistical analysis of their consumption could not contribute to explain the comparably low concentrations of plasma lycopene in the AN cohort. Besides eating habits, seasonal effects of fruit and vegetable consumption cannot be excluded.

Elevated plasma carotene levels have previously been observed in AN patients (26, 27) and are closely connected to the development of hypercarotenemia (28, 29). Hypercarotenemia occasionally occurs in AN patients (13, 28, 30, 31). However, the underlying cause of this occurrence is not completely understood yet (27). Still, some possible mechanisms have been described:

It is hypothesized that the higher plasma concentrations and the accumulation of carotenoids in skin tissue may be attributed to the high consumption of carotenoid-rich food such as fruits and vegetables in AN patients. AN patients commonly prefer food low in energy. Thus, fruits and vegetables may be consumed in unusually high amounts from AN patients compared to their total energy intake (11, 12). Our study could not confirm this hypothesis since fruits and vegetable consumption was comparable to NW controls. However, when adjusted for SAT, AN patients consumed more fruits and vegetables in relation to their body fat amount.

Secondly, a possible explanation for elevated β-carotene levels in AN patients is proposed by the strong connection of lipid metabolism and the appropriate degradation of carotenoids (14). Disturbances in lipid metabolism are often reported in AN patients. (13, 27, 32, 33). Additionally, certain diseases such as hypothyroidism and diabetes mellitus have been associated with altered carotenoid degradation (3, 27). However, the AN cohort investigated in this study had no striking plasma lipid values, and also fasting glucose and thyroid markers were within normal ranges.

Third, the amount of body fat has been suggested to be crucial for the concentration of circulating carotenoids (34). High plasma carotenoid levels may be a marker of a systemic carotenoid overload mainly because of low storing ability in fat mass.

### Lower Body Fat Is Associated With Higher Carotene Plasma Concentrations in AN Patients

A highly significant correlation of plasma β-carotene concentrations and subcutaneous body fat was observed for the whole study population. However, this correlation was even higher for the AN patients alone. Moreover, also β-cryptoxanthin and the total amount of provitamin A carotenoids were negatively correlated to body fat in the AN group. Still, they did not show a correlation in the NW group. This observation could contribute to the following mechanism:

Carotenoids are stored and processed in adipose tissue. More carotenoids remain in the circulation or are stored alternatively in other tissues such as the skin if the fat depots are depleted (34, 35). Recently, we have shown that AN patients significantly differ according to body composition and subcutaneous fat despite their low BMI (24). About half of the patients had SAT values comparable to normal healthy controls. This occurrence can also be seen in Figure 2. In addition to the negative correlation of carotenoids and SAT in the AN patients group, significant differences in carotene levels within the two AN body composition groups could be observed. The AN group with the low SAT amount had significantly higher plasma and skin carotenoid concentrations than those with more SAT (36). Thus, the association of body fat and β-carotene concentrations is likely.

In total, our observation supports the third hypothesis for the occurrence of hypercarotenemia. Marked differences in plasma carotenoid concentrations in AN patients have been associated with SAT thickness levels. Additionally, fruits and vegetable consumption relative to SAT thicknesses were significantly elevated. On the contrary, Robboy et al. found a decrease in serum β-carotene levels in cachectic AN patients (37). Noteworthy, this observation was based solely on data of eight AN patients. Interestingly, hypothalamic amenorrhea, linked to reduced body fat storage, leptin levels and energy availability, has also been associated with hypercarotenemia (38).

### Elevated Plasma Carotenoid Levels May Alter Weight Gain and Recovery of Depleted Body Fat Stores

The role of carotenoids in adipocyte biology has been described for adipocyte's fat storage capacity and adipogenesis (8, 9, 25). β-carotene, is an important retinoic acid precursor. It acts as a nuclear factor in adipocytes and contributes to the modulation of fat storage capacity, reduction of oxidative stress, elevates fat depletion and contributes to enhanced thermogenesis (39). Moreover, it enables preadipocytes to mature and thus influences adipocyte differentiation and the amount of adipocytes available (7). Taken together, carotenoids are therefore suggested to be beneficial in obese metabolism, which is also supported by data of cell culture and animal models (40) and human data (8, 9).

On the contrary, it could be possible that the opposite is the case for AN patients. As a consequence of the high fat turnover induced by high carotenoid levels, it may be possible that the high β-carotene levels observed in AN patients limit fat storage and thus weight gain.

We hypothesize that high β-carotene levels that have been observed in AN patients with extremely low amounts of body fat may inhibit body fat storage and thus sufficient weight restoration. To our best knowledge, there are currently no clinical human studies investigating the role of carotenoids in undernutrition and weight gain. Therefore, further research on the possible inhibiting effects of elevated carotenoid levels on the recovery of AN patients and the recovery of body fat stores is warranted. Further studies should test this hypothesis in longitudinal settings. Additionally, studies to explore the mechanisms of carotenoids on weight gain and strategies to counteract these potential inhibitory effects are needed.

## Limitations

Last but not least some limitations of the current study need to be emphasized. The anti-obesity effects of carotenoids are primarily exerted by their conversion compounds. Provitamin A carotenoids are cleaved to retinoids. The anti-obesogenic effect of carotenoids is best described for the nuclear function of all-trans-retinoic acid (atRA), however, also other retinoids and non-retinoid apocarotenoids have been shown to interact with nuclear receptors (10). Further investigations need to consider the determination of plasma retinoids including atRA to further explore the observed associations of carotenoids and body fat.

The data presented here is accociative only, causality cannot be inferred, and the number of Anorexia nervosa patients investigated here is limited. Thus, further research is needed to validate the hypotheses raised here in larger cohorts.

## Conclusion

The antiobesity effects of carotenoids may have an important impact on the pathophysiology and treatment of AN. Our data support a possible effect of a systemic carotenoid overload on changes in adipogenesis and metabolic capacities for energy storage.

We showed that higher plasma β-carotene levels were likely to be associated with reduced body fat levels, most probably due to a decreased ability of the remaining adipose tissue to store carotenoids. This phenomenon may inhibit the organisms' ability to restore fat appropriately at the same time. As a consequence, therapeutic efforts may be mitigated. Further studies need to prove this hypothesis in a longitudinal setting.

## Data Availability Statement

The raw data supporting the conclusions of this article will be made available by the authors, without undue reservation.

## Ethics Statement

The studies involving human participants were reviewed and approved by the Ethics Committee of the Medical University of Graz. The patients/participants provided their written informed consent to participate in this study.

## Author Contributions

SH, SL, and SM designed the study. SL, SH, NM-A, and SM wrote the manuscript. SL and NM-A calculated the statistics. NM-A designed the figures. SL collected and analyzed nutritional data. SL, SM, AF-R, and WM performed the ultrasound analysis. HM and SZ analyzed and interpreted blood samples. NM-A performed HPLC analysis. All authors contributed to the article and approved the submitted version.

## Conflict of Interest

The authors declare that the research was conducted in the absence of any commercial or financial relationships that could be construed as a potential conflict of interest.

## Publisher's Note

All claims expressed in this article are solely those of the authors and do not necessarily represent those of their affiliated organizations, or those of the publisher, the editors and the reviewers. Any product that may be evaluated in this article, or claim that may be made by its manufacturer, is not guaranteed or endorsed by the publisher.

## Abbreviations

AN: anorexia nervosa
atRA: all-trans-retinoic acid
BMI: body mass inde
D_INCL_: sum of subcutaneous adipose tissue thicknesses measured at the eight standardized body sites
ICD: international classification of diseases
IQR: interquartile range
NW: normal weight
SAT: suncutaneous adipose tissue
WHO: world health organization.
